# Governing therapeutic pluralism: An environmental scan of the statutory regulation and government reimbursement of traditional and complementary medicine practitioners in the United States

**DOI:** 10.1371/journal.pgph.0001996

**Published:** 2023-08-09

**Authors:** Nadine Ijaz, Heather Carrie

**Affiliations:** 1 Department of Law and Legal Studies, Faculty of Public Affairs, Carleton University, Ottawa, Canada; 2 Heather Carrie Research Associates, Vashon Island, Vashon, Washington, United States of America; Jawaharlal Nehru Medical College, INDIA

## Abstract

The World Health Organization has called on nation-states to statutorily govern, and integrate into state-funded healthcare systems, practitioners of traditional and complementary medicine (T&CM) (whose therapeutic approaches that fall outside the boundaries of conventional biomedicine). To date, however, there exist few rigorous reports of the degree to which individual nations have responded to this call. This study, an environmental scan, comprehensively documents the statutory governance and government reimbursement of T&CM practitioners in the United States (US). Across the US, where health practitioner governance falls within state and territorial (rather than federal) jurisdiction, over 300 laws have been enacted to statutorily regulate a wide range of T&CM practitioners. Nurse-midwives and chiropractors are universally licensed across all 56 US regulatory jurisdictions (50 states, 5 territories and the District of Columbia); other major T&CM practitioner groups are regulated in fewer jurisdictions (acupuncturists, n = 52; massage therapists, n = 50; direct-entry [non-nurse] midwives, n = 36; naturopaths, n = 24). Additional statutory stipulations exist to govern chiropractic assistants (n = 30), auricular (ear) acupuncture practitioners (n = 24), homeopathic practitioners (n = 3), and psychedelic facilitators (n = 1), as well as biomedical professionals who practice acupuncture and related techniques, e.g., ‘dry needling’ (n = 44). While professional entry requirements for licensed T&CM practitioners are substantially harmonized across jurisdictions, restricted titles and statutory scopes of practice vary. Ten states have furthermore implemented ‘safe harbor’ (‘negative licensing’) exemption laws enabling otherwise-unregulated T&CM practitioners to legally practice. Limited government reimbursement for T&CM care is available across several federal and state programs, including Medicare, Tricare, Veterans Health Authority, and Medicaid. Nurse-midwifery and chiropractic care is most frequently reimbursed; acupuncturists, naturopaths and massage therapists are eligible for much more limited coverage. Medicaid programs for low-income people in ten states furthermore cover the services of (unlicensed but statutorily-recognized) birth doulas. Additional research is needed to assess the impact of these regulations on US health care.

## Introduction

Since the World Health Organization (WHO)’s publication of its first Traditional Medicine Strategy in 2002, governments worldwide have increasingly taken up the WHO’s repeated calls for increased state governance of traditional and complementary medicine (T&CM) [1]. T&CM refers to a wide range of therapeutic practices and products that fall outside of the boundaries of conventional Western biomedicine, a therapeutic system sanctioned by governments worldwide [1–3]. T&CM therapeutics—the primary form of accessible healthcare in many Global South nations, and in widespread demand across the North—include pre-colonial Indigenous healthcare approaches (e.g., Ayurveda Chinese medicine, acupuncture, ceremonial healing practices), as well as more recently-developed, unconventional therapeutics (e.g., naturopathy, chiropractic, homeopathy). The WHO’s call for increased statutory governance of T&CM practitioners strives toward greater safety, quality and accessibility of such therapies. The call includes three primary elements: the regulation of products and practitioners, and the inclusion of T&CM therapies into state-funded health systems. In 2018, 78 of the WHO’s 194 member states self-reported implementation of T&CM practitioner-related governance strategies [3]. (The governance of T&CM-related products such as herbal medicines and natural health products falls outside the present work’s purview.)

Worldwide, T&CM plays an “important role in meeting …primary health care needs”, particularly in global South countries and, across the North, is widely used as a complement to conventional biomedical care, particularly for treating complex, chronic conditions [2]. In addition, traditional medicine approaches represent an important source of culturally-relevant healthcare, both in their geographies of origin, and in diaspora. In that the principle of T&CM’s health systems integration “uses the resources, knowledge, and experience of diverse societies to address health challenges throughout the world,” it may be understood to be an issue that falls within the purview of global health studies [4].

Across nations, T&CM practitioners may include, in various permutations: conventional biomedical healthcare providers trained in specific forms of T&CM (e.g., medical doctors or nurses trained in acupuncture); non-biomedical clinicians trained formally in a particular area of T&CM (e.g., practitioners of Ayurveda, naturopathy, East Asian medicine, chiropractic, osteopathy, or homeopathy); and, persons (including Indigenous healing practitioners) trained informally (e.g., through apprenticeship and/or family lineage), or through a combination of formal and informal training in T&CM therapeutic approaches. As elsewhere observed, the statutory governance and reimbursement of T&CM practitioners raises a range of distinct policy making challenges. These include: how to appropriately structure licensure frameworks for occupations whose clinical epistemologies may diverge from biomedical science, and may not be internally standardized; how to statutorily contend with the concurrently clinical and cultural dimensions of traditional medicine practices; and what evidentiary standards may provide an appropriate basis for T&CM reimbursement decisions within a biomedically-dominant context in which conventional clinical trial methodologies may not be sufficient for appropriate for evaluating T&CM therapeutics [5].

Although there is a need for additional conceptual scholarship in this area, the WHO provides a useful typology of national health care systems with respect to T&CM practitioner governance and integration [5]. This typology characterizes government health systems as a spectrum characterized by four primary categories: a) biomedically ‘exclusive’ (where T&CM is outlawed); b) ‘tolerant’ (in which some T&CM practices are legally permitted but poorly integrated into health systems); c) ‘inclusive’ (characterized by partial regulation and/or integration of T&CM care into public health systems); and, d) ‘integrative’ (in which both biomedical and T&CM care are legally recognized and funded). This spectrum allows for a range of governance approaches, both in terms of what types of personnel may be delivering T&CM care, and the degree to which (and contexts within which) such care may be reimbursed by the state.

‘Integrative’ nations (e.g., China, South Korea, Vietnam) typically include T&CM-focused stipulations in their constitutions or related national policies; offer T&CM-related university-level education for biomedical and state-sanctioned T&CM specialist professionals; and, extensively reimburse T&CM within public healthcare settings. ‘Inclusive’ countries (e.g., India, Canada, Germany, United States), by contrast, may vary considerably in terms of national T&CM policy implementation, statutory regulation of T&CM practitioners (and related educational strategies), and integration and reimbursement of T&CM in public healthcare settings. Elsewhere, in countries better characterized as ‘tolerant’(e.g., France), it may be biomedical health professionals alone who are legally permitted to practice T&CM approaches (with or without state reimbursement), and non-biomedical T&CM practitioners may be largely outlawed or implicitly tolerated to practice within legal grey areas [6].

Despite a slow but progressive increase in the number of nations that have implemented statutory governance of T&CM practitioners over the last two decades, 96% of WHO member-states identify a “lack of research data” as the primary barrier faced in enacting T&CM-focused policies [2]. Globally, as Ijaz and Boon observe, there continues to be a scarcity of rigorous, synthetic resources characterizing key T&CM governance issues [5]. While the WHO has published brief T&CM policy profiles for most member-states, these include no source citations, and—in several cases, such as with respect to the United States (US), the current study’s focus—are notably incomplete [3].

As shown in the present work, the US is a country with extensive statutory governance of T&CM health care practitioners, enacted through over three hundred pieces of legislation, as well as numerous examples of government reimbursement for T&CM therapeutics. However, the WHO’s 2019 Traditional Medicine Report, which relies on data voluntarily disclosed by federal governments in its reporting, minimally indicates with respect to T&CM practitioner governance in the US that “[r]egulations for T&CM providers are delegated to each of the 50 states”; and incorrectly reports that it is only “private health insurance” programs that reimburse T&CM care across the country [3]. To address this notable gap, our study uses an environmental scan methodology to rigorously document the statutory governance and government reimbursement picture for T&CM practitioners and practices across the US. Before turning to the study methods, we provide an overview of the broader context of health professional governance and reimbursement across the US, with reference to prior studies addressing T&CM practitioners and practices.

### The broader context of T&CM governance in the United States

As the present study will illustrate, the US health care system would best be characterized as ‘inclusive’, in that: many T&CM practices are explicitly or implicitly considered legal; b) several T&CM occupational groups (including chiropractors, homebirth midwives, acupuncturists, naturopaths, and massage therapists) are subject to statutory licensure; c) formal medical specialty areas have been implemented for biomedical doctors and nurses with training in T&CM approaches; and, d) there exists a minor degree of state health funding for T&CM services, as well as some statutory provisions requiring that private (commercial) insurance companies reimburse such services. Before turning to the study methods, we provide a contextual overview of prior scholarship and grey literature reporting in these areas.

#### A primer on health practitioner governance in the US

In the US, health practitioner governance falls within state (rather than federal) jurisdiction. While laws governing health care workers today vary from state to state, all states have medical licensing regimes that legally constitute the practice of medicine “using such words as *diagnosis*, *treatment*, *prevention*, *cure* and *prescribe”* [7]. Across the US, the practice of medicine without a license is generally constituted as a crime; and US law entrenches conventional biomedicine as a normative reference point around which all therapeutic practices are governed. Several statutory modes of health practitioner governance have been implemented in the US, including: limited and unlimited licensure, mandatory licensure, title protection, statutory registration, certification, and licensing exemptions (negative licensing).

Biomedical physicians (MDs and DOs) are granted *unlimited* licensure across the country, granting them “broad leeway to diagnose and treat disease” [7]. Other health professionals, whether trained in biomedical and/or T&CM therapeutics—may also be subject to state licensing laws, but are generally granted *limited* licensure, authorizing “a narrower scope of practice” than biomedical physicians. *Mandatory licensure*, a construct entrenched in some health professional licensing statutes, explicitly stipulates that “an individual cannot practice without a state license”, often at peril of criminal prosecution [7]. Some states, conversely, use mechanisms of *licensing exemptions*, also referred to as *negative licensing*, to explicitly exclude certain practices that might otherwise be construed as falling under the scope of medical practice (e.g., bodily piercings, reflexology, massage therapy), leaving such practices in the public domain or carving out terrain for their governance by other means [7, 8]. Many health professional licensing laws also implement *statutory title protection*, whereby members of a particular licensed health profession have exclusive authority over particular the use of professional titles (e.g., Registered Nurse) and even the public use of particular language describing the work they do (e.g., nursing) [7]. Some states implement *statutory registration*, which in some states is a term synonymous with *professional licensure;* elsewhere, however, it may refer to a legal requirement for otherwise-unlicensed practitioners of a specified therapeutic approach to disclose, to a state agency, information about their qualifications to practice. Related, some states mandate that practitioners of specified therapeutic approaches document *certification* with a defined external agency but do not implement licensure for such practitioners. We endeavor to specify with clarity which of the aforementioned range of state governance approaches are being characterized in the study findings.

Professional entry into licensed health profession in the US is typically contingent on completion of uniform educational requirements stipulated in state statutes, rules and/or regulations. Such requirements (like statutory scopes of practice) may vary from state to state, even for a single occupation. In many cases, state regulators accredit or recognize particular educational institutions, or recognize the authority of educational accrediting bodies external to the state. State regulators may also sanction the authority of external (non-state) certification bodies to confirm practitioner qualifications for state licensure.

To better understand the statutory context of T&CM practitioner governance in the US, it is important to be aware of several T&CM-related contextual factors that may differentiate the US from other jurisdictions, in particular with respect to biomedical health professionals’ relationship to T&CM care. In particular, a group of laws exist in more than thirty US states that align with a principle dating back to the late 1800s, called ‘the Corporate Practice of Medicine (CPOM) Doctrine’. Such laws are designed to differentiate ‘regular’ biomedical physicians from so-called ‘irregulars’ (that is, T&CM practitioners), and include mechanisms that “prevent regular physicians from practicing with irregular practitioners” [9]. For example some states preclude medical doctors from being employed by chiropractors, or prevent chiropractors from “having direct ownership interests in medical practices” [9]. Although the full impact of CPOM Doctrine is beyond the present work’s scope, we provide an overview, in what follows, of a range of the degree of institutionalization of T&CM approaches within key conventional biomedical professions, and of other key elements of the T&CM regulatory and reimbursement landscape in the US today.

#### Biomedical doctors

In the US, there exists a subset of conventional biomedical physicians—widely referred to as ‘integrative’ physicians—who variously include T&CM therapeutic approaches in their clinical work. It is important to note that in the US, the category of conventional biomedical physician includes, by definition, two distinct occupations: licensed ‘medical doctors’ (MDs) and ‘licensed osteopathic physicians’, that is, doctors of osteopathy (DOs). While the two groups were historically distinct, and continue to be educated at different institutions, their licensure is considered legally equivalent across the country [10, 11]. Although ‘osteopathic manual therapies’ (considered by the WHO to be a T&CM approach) continue to be taught in US medical schools that train DOs, these therapies are no longer widely-used by such physicians [12]. As such, the DO credential in the US should not be conflated with statutory recognition of osteopathic manual medicine practitioners, who are not otherwise subject to licensure in the US today; furthermore, licensed DOs should not be presumed to necessarily practice in an ‘integrative’ manner.

In 2014, the American Board of Physician Specialties—one of two major specialty-granting bodies for MDs and DOs across the US—recognized ‘integrative medicine’ as a new specialty, which continues to be governed under the auspices of the American Board of Integrative Medicine [13]. Eligible integrative medicine specialists must be licensed MDs or DOs who have additionally completed defined training in complementary and integrative medicine, as well as a standardized examination. However, the legal position of individual MD/DO’s who wish to incorporate unconventional therapies into clinical practice rests on interpretation of individual state medical practice acts, as well as related policies and practice standards.

We are unaware of any prior analysis of state policies or practice standards with respect to conventional doctors’ integration of T&CM therapies into clinical practice. However, in 2002, the US Federation of State Medical Boards published a policy document entitled, ‘Model Guidelines for the Use of Complementary and Alternative Therapies in Medical Practice’ [13]. This policy, meant as a template for state medical boards, both “allow[s] a wide degree of latitude in physicians’ exercise of their professional judgment” and recognizes patients’ “right to seek any kind of care for their health problems”. The policy emphasizes that unconventional treatments offered by physicians should “have a favorable risk/benefit ratio compared to other treatments for the same condition; be based on a reasonable expectation . . .[of] a favorable patient outcome” and be expected to yield “greater benefit . . .than . . .no treatment” [14].

As detailed further on in the present work, there are some areas of T&CM practice (e.g., acupuncture, homeopathy) for which particular state regulators for MD/DOs have made explicit statutory stipulations.

#### Nurses

In the US, licensed nurses who incorporate T&CM approaches into their work are widely referred to as ‘holistic nurses’. Since 2006, the American Nurses Association—the nation’s primary professional nursing association—has recognized Holistic Nursing as a distinct nursing specialty. The specialty is accompanied by a specified scope and practice standards, which includes several T&CM health practices (e.g., meditation, yoga, energy therapies [14]). Another nursing specialty recognized by the American Nurses Association with some overlap with holistic nursing is known as ‘faith community nursing’ [15]. However, individual nurses must adhere to the scope of practice stipulations articulated in their state nursing practice acts. Stipulations related to the practice of holistic nursing vary by state. According to a 2021 analysis conducted by the American Holistic Nursing Association [16], seventeen US states including explicit reference to holistic nursing practices in their nursing practice legislation and/or related policy documents. While we will not elaborate further on these points here, we note further on in the present work that some state laws include acupuncture within the scope of advanced nursing professionals (‘nurse practitioners’), who receive additional graduate level training in nursing. In addition, as detailed further on, nurses with additional childbirth-related training may be licensed as nurse-midwives across all US states.

#### Dentists

Although some US dentists incorporate T&CM therapeutic approaches into their clinical practices, and/or practice in ways otherwise distinct from their profession’s standard of practice, the US profession of dentistry does not currently recognize holistic or integrative dentistry as a specialty field. In 2011, the American Dental Association released a policy statement on complementary and alternative medicine in dentistry, which characterizes “interventions which are considered CAM” as “understudied interventions that require further scientific testing” [17]. In that same statement, the Association characterizes “the notion of CAM as a specific subset of interventions that belong to a specific discipline” as “questionable”.

#### Prior US-focused scholarship about T&CM practitioner governance

Several scholars have previously reported on particular aspects of T&CM practitioner governance across the US, including studies addressing the overall regulatory contexts for such practitioners in the late 1990s and early 2000s [17]. Herman and Coulter have, more recently, provided an overview of the complex T&CM policy landscape in the US, pointing to tensions that arise as a result of differential licensure patterns across states and, in particular, whether T&CM practice is defined in policy as a ‘modality’ (e.g., acupuncture, massage therapy) or as part of the scope of a broader profession (e.g., acupuncturist, massage therapist) [18–20]. We discuss this issue again further on with respect to the context for T&CM reimbursement in the US.

In addition, a few recent studies specifically address the US context of acupuncture governance, including with respect to: statutory provisions governing licensed acupuncturists trained in East Asian medicine approaches, acupuncture-practising biomedical health care professionals [10], and providers of auricular (ear) acupuncture [21]; as well as studies characterizing the US acupuncturist workforce context, pointing both to significant growth within that occupational group over the last two decades [22], significant differences in practice acts by state, and to the current absence of rigorous related demographic and income data [23]. Other scholarly works address the historical and contemporary contexts of midwifery governance; a growing trend toward statutory governance and reimbursement of birth doulas [21]; chiropractic credentialing and scopes of practice [24]; licensed massage therapists’ vulnerability to sexual harassment and solicitation within a broader context wherein sex trafficking operations misrepresent themselves as massage therapy businesses [24] ; the potential for naturopathic doctors working as primary care providers to reduce health professional shortages [25]; legal issues surrounding medical doctors’ use of T&CM practices; and, ‘safe harbor’ state laws enabling the limited practice of T&CM therapies by unlicensed practitioners [26]. A few studies furthermore point to a broader context of unlicensed T&CM practitioners offering care across the US [27], whether as herbalists, yoga therapists, mindfulness educators, Ayurvedic practitioners or faith healers ; in the form of bodywork practices that fall outside of massage therapy’s legal boundaries [28]; or, with respect to the wide range of traditional/Indigenous therapeutic practitioners who offer care within ethno-specific communities [29]; at times in collaboration with biomedical professionals [30].

In addition to the aforementioned studies, grey-literature and other online reports by non-governmental organizations have addressed, in various formats, the legislative standing and/or reimbursement contexts of particular T&CM occupational groups, including acupuncturists [19, 31–40], birth workers (e.g., midwives and doulas ) [38, 41–49], chiropractors [50–61], homeopaths [62], massage therapists and bodyworkers [60, 63–75], naturopaths [56, 76–82], and psychedelic facilitators [83–86], as well as otherwise-unlicensed practitioners [e.g., 87]. To date, however, there has been no comprehensive account of the overall picture of statutory governance with respect to T&CM practitioners in the US, one of the gaps the present work aims to address.

The present study’s other major aim is to provide an overview of government reimbursement, and/or government-mandated reimbursement of T&CM services in the US, another area where prior scholarship remains patchy. Before turning to the study methods, we provide readers with an overview of the US health care reimbursement context, with reference to related scholarship addressing T&CM care.

### Government-subsidized care and third-party health insurance reimbursement in the US

**Fig 1:**
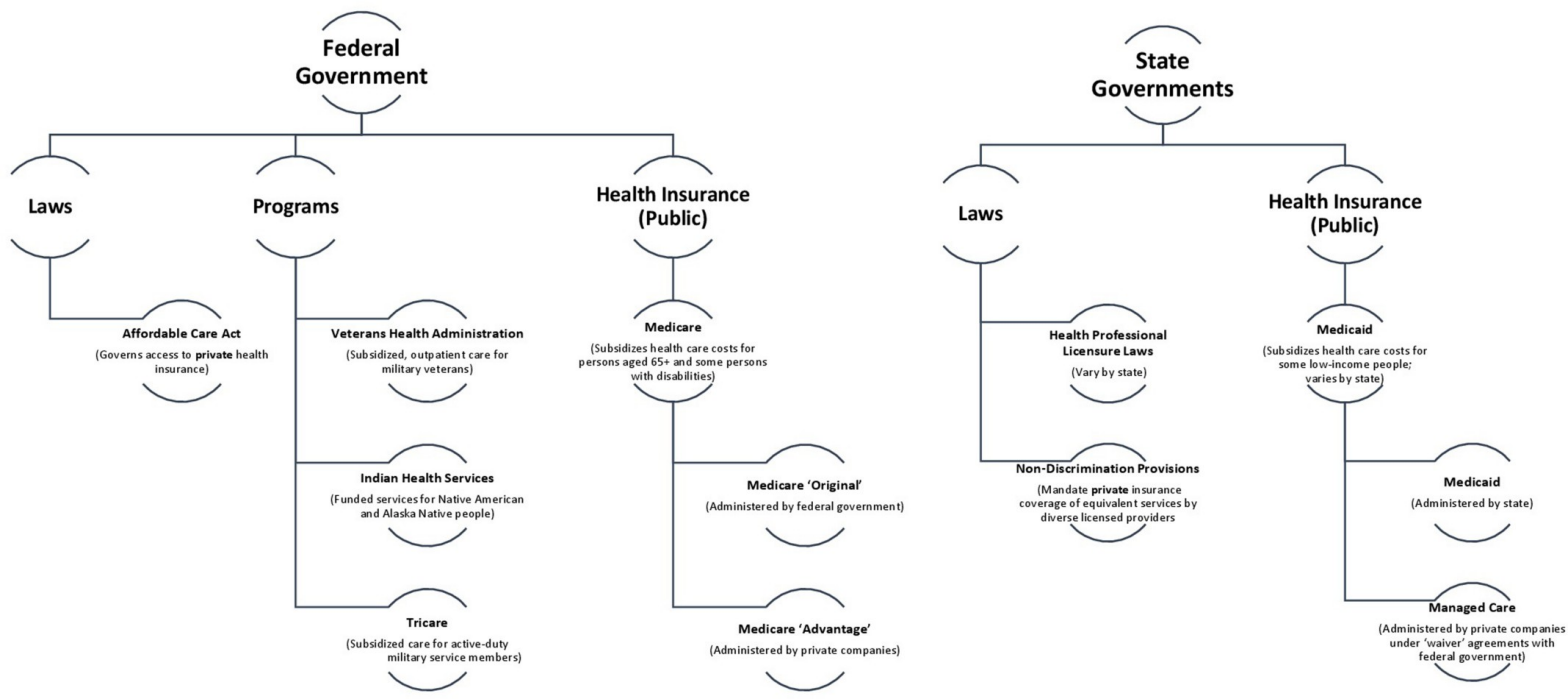
Federal and state governance and reimbursement of health care in the United States.

The US is a country without a universal (‘single-payer’) government-funded health insurance program universal a ‘single health care payer’. Instead, health care costs are largely paid either out of pocket, through a third-party insurance program, or a combination of the two. In the US, third-party payers may be federal or state government agencies, private companies, non-profit organizations and/or employers; further, the reimbursement patterns of commercial payers are at times determined by statutory stipulations. Below, we provide an overview of the country’s publicly-funded health care reimbursement context, as well as key statutory provisions that mandate particular coverage patterns by private payers. Fig 1 offers a visual map of the health-related laws, programs and government insurance programs that will be further discussed in the present work.

#### Publicly-funded health care in the US

There are five major programs in the US under which government funds subsidize health care costs: Medicare, Medicaid, Indian Health Service, Tricare, and the Veteran’s Health Administration. Each has its own associated set of reimbursable services, payment calculation approaches, and reimbursement mechanisms.

Medicare and Medicaid are health insurance programs meant to support access to health care by members of particular vulnerable populations. *Medicare* is a federal government health insurance program for persons aged 65 and older, and some persons with disabilities, offering coverage for hospital-based care, a defined set of outpatient medical services and supplies, and the cost of prescription drugs. Medicare beneficiaries pay monthly premiums to retain access to certain aspects of their coverage, also contributing ‘co-payments’ or ‘deductibles’ for some insured services. Medicare recipients may choose to access their benefits directly from the federal government (‘Original Medicare’) or through a Medicare-approved plan implemented by a private company (‘Medicare Advantage’) [88]. *Medicaid*, a health insurance program administered at the state (rather than federal) level, which subsidizes health care costs for some low-income people. Currently Medicaid programs are in place in all states. Medicaid benefits may be delivered directly by state governments or through ‘Managed Care’ programs in which, under ‘waiver’ agreements with the federal government, private insurance companies (who receive monthly premiums from state governments) deliver Medicaid benefits directly to eligible beneficiaries. While federal law mandates some health care services that must be covered by state Medicaid programs (e.g., hospital, physician and laboratory services), other benefits are optional (e.g., prescription drug coverage, dental and vision care). Medicaid benefits thus vary by state [89].

The US government additionally funds three distinct health programs—*Indian Health Services*, *Tricare*, and the *Veterans Health Administration—*each of which exclusively serves a particular US sub-population and has a defined set of ‘funded’ health services. *Indian Health Services*, administered as a division of the US Department of Health and Human Services, provides healthcare free of charge to Native American and Alaska Native people across the country, through a network of hospitals and outpatient centers. Eligible persons may receive inpatient and outpatient care for a defined set of services within a network of Indian Health Service and tribal medical facilities [90]. *Tricare* is a federal health care program, operated by the Department of Defense, which subsidizes specified civilian health and dental care and prescription drug costs for active-duty military service members, National Guard and National Reserve members, retirees, and their families [91]. With respect to care defined as medically-necessary, many beneficiaries are subject to an annual ‘deductible’, a co-pay portion for some services or prescriptions, and in some cases, an annual enrollment fee and/or monthly premium. The *Veterans Health Administration* is a national healthcare program that delivers outpatient care to US military veterans at a network of Veterans Affairs hospitals and outpatient clinics staffed by government employees. Eligible veterans may pay ‘co-payments’ (but no premiums or deductibles) for a portion of some kinds of eligible care costs.

A few previous studies address government reimbursement of T&CM care through the aforementioned programs. One study observes that chiropractors—who are constituted as physicians under Medicare laws—are the only licensed T&CM profession in the US to be eligible for direct reimbursement by Medicare; “an act of Congress” would be required to directly reimburse other T&CM professionals. Similarly, “some state Medicaid systems limit coverage to the provider types listed by Medicare”[9].

Other research addresses a recent trend toward coverage of non-pharmacological pain treatments (including various T&CM services) in the context of the ongoing opioid crisis [92–95]. Two recent studies provide detailed nationwide analyses of existing government reimbursement of non-pharmacological pain treatments; these works document a high degree of heterogeneity with respect to T&CM coverage across states and within federal programs [92, 96]. T&CM services covered by Medicaid in some but not all states and/or in some federal government programs include spinal manipulation, acupuncture, massage therapy, mindfulness and meditation, t’ai chi / qi gong, and yoga. Two other studies specifically report on the impacts of the state of Oregon’s inclusion of T&CM services for back pain within state Medicaid plans. As in prior studies documenting a connection between private insurance coverage for T&CM care and usage of such services [97, 98], Choo and colleagues observed [93, 95] an increase in T&CM service uptake among Oregonians following Medicaid inclusion [95]. However, uptake was not even across all demographics, with heightened increases observed among female, urban and older populations (and lesser uptake among “Black, American Indian / Alaska Native, and Hispanic [Medicaid] enrollees” [95].

It is important to note that the Veterans Health Affairs network include eighteen ‘Whole Health’ flagship sites across the country, along with thirty additional ‘Whole Health’ design sites, all “focused on . . .a personalized health plan that considers the physical, mental, emotional, spiritual and environmental needs of Veterans”. ‘Complementary and Integrative health’ is constituted as a central aspect of the Whole Health model, with some T&CM professionals being hired on a salaried basis at Veterans Health Affairs sites [9]. In addition, some Veterans Affairs sites include ‘healing lodges’, where Native American traditional healing ceremonies (e.g., sweat lodges, talking circles) take place for veterans; at least one such site was purpose-built using government funds [99, 100].

#### Statutory provisions for commercial health insurance

Statutory provisions have been implemented both at the US state and federal levels that require private insurers, in particular cases, to reimburse T&CM practices and/or practitioners. These include two primary mechanisms: non-discrimination provisions, and essential health benefits.

As Herman and Coulter have observed, the reimbursement landscape for T&CM care in the US is rendered complex by the fact that third-party insurers may reimburse for particular ‘modalities’ (e.g., massage therapy, acupuncture) but not always recognize particular professional groups (licensed massage therapists, acupuncturists) as eligible providers of the services they are qualified to offer [9]. Non-discrimination provisions statutorily mandate commercial insurance companies to ‘not discriminate’ between licensed health care providers when reimbursing for particular insurable services. In 1996, for example, the state of Washington introduced a non-discrimination provision requiring private health insurance companies to cover services determined to be insurable, whether delivered by by biomedical professionals or by licensed T&CM professionals (e.g., chiropractors, acupuncturists, naturopathic doctors, massage therapists) [101–104]. Similarly, over forty states require that private insurers cover chiropractic care, at least eight have required reimbursement of licensed acupuncturists, and at least one additional state requires coverage of naturopathic care [103–105]. Further, in 2014, non-discrimination provisions were introduced under section 2706 of the federal Affordable Care Act [106]; these provisions aimed to prohibit private insurers from “discriminating against [T&CM] providers who are acting within the scope of their licenses” with respect to similar services otherwise reimbursed for conventional biomedical providers. However, implementation of the federal non-discrimination provision has been uneven across states [107], owing to the provision having been “written in such a way that may limit its impact on coverage decisions” [9]. Further, even where non-discrimination provisions have been implemented at the state level, many insurance companies continue to reimburse differentially between different types of providers (e.g., paying medical doctors more than chiropractors for the same service) [103, 107, 108].

Another type of statutory provision potentially impacting coverage of T&CM services involves ‘Essential Health Benefits’ (EHB), which refer to a “basic set of insurance benefits” included in private health insurance plans under a mandate under the Affordable Care Act [94]. In a state-by-state analysis of the inclusion of non-pharmacological treatments for low back pain within EHB benchmarks showed that spinal manipulation for low back pain (e.g., chiropractic or osteopathy) was included as an EHB in forty-six states. Hopes for increased T&CM coverage under EHB requirements were high when the Affordable Care Act (which has been since been modified in various ways) was first introduced [109]. However, despite a 2017 evidence-based guideline released by the American College of Physicians that recommended use of several T&CM approaches for low back pain (e.g., acupuncture, massage, yoga, tai chi), such have to date been included in “fewer than 10 states’ benchmarks plans”, often with “caps . . .placed on the number of treatments” [96].

## Methods

The current study, an environmental scan, addresses the question: *In the United States*, *how are practitioners of unconventional (i*.*e*., *‘traditional and complementary medicine) therapeutics statutorily governed and reimbursed by government programs*?

### Methodological overview

Environmental scans serve to map the terrain of a given sector or field of interest, including where related scholarly literature is lacking. As a method with origins in the business sector, environmental scans are increasingly used by scholars (including in the health and policy-making fields) [110–112]. Methodological literature on environmental scans is limited, and guidance varies across sectors, but such scans share several guiding characteristics [111–114]. Environmental scans typically begin with identification of a driving question and a definition of this question’s scope, which may be iteratively nuanced as the project unfolds. Data generation in environmental scans typically draws on multiple source types, including grey and scholarly literature searches, surveys, interviews and other approaches specific to the area under study. Inclusion and/or exclusion criteria for particular data types may evolve over the course of data collection, in tandem with clarification of the scan’s driving question. Analytic techniques may be qualitative and/or quantitative, focused on development of a comprehensive account of the sector under study, as well as identification of relevant trends. Findings from environmental scans may inform needs assessments, strategic planning, program design and development, decision making, and further research.

### Study scope

With respect to the study’s driving question, the study authors constitute our inquiry’s scope to primarily address those occupational groups and therapeutic practices nationally and internationally constituted as ‘unconventional’, ‘non-biomedical’, and/or ‘traditional and complementary’, in the sense that they are not typically included as core treatment modes in biomedical professional training programs. We do not endeavor to provide specific definitions or descriptions of such occupations as these are widely available elsewhere (e.g., [2, 115]). Although it is contested whether midwifery should be constituted as a T&CM occupation, we include it (along with the work of other non-biomedical birthing personnel, such as doulas) within the present work’s scope, since it represents—as elsewhere argued [115]—a form of birthing care distinct from conventional biomedical obstetrics. In addition, we include the relatively-new occupation of psychedelic facilitation in our analysis, given that this is (like acupuncture or yoga) a traditional Indigenous therapeutic practice that is increasingly entering the mainstream, giving rise to distinct policy questions. Such questions include the degree to which the facilitated, therapeutic use of psychedelics might belong in the public domain, should be restricted to being an Indigenous ceremonial or religious practice, and/or warrants inclusion in the clinical toolkits of biomedically-trained professionals [116–120]. Our study also includes within its scope explicit statutory provisions with respect to the use of unconventional (T&CM) therapies by conventional biomedical health practitioners’ (e.g., medical doctors, nurses); but, we do not treat in any depth non-statutory policy documents that may interpret such statutory provisions.

Jurisdictionally, the study’s scope addresses US federal government policy, as well as governance at the level of the state, district or territory (i.e., across each of the fifty states, the District of Columbia (DC), and the five territories (Puerto Rico, Northern Mariana Islands, US Virgin Islands, Guam, and American Samoa). This work does not generally provide a review of municipal government policies.

Within the present study’s context, we define *statutory governance* as a broad construct that includes: statutory regulation and other legal governance of various kinds: practitioner licensure; occupational title protection; state registration; voluntary or mandatory state certification of practitioners; as well as regulatory provisions, exemption laws (‘negative licensing’) and, in a few cases, court rulings addressing scope of practice. Modes of non-statutory governance (e.g., voluntary self-regulation, certification by non-governmental bodies) fall outside the present work’s purview except where linked to state governance. The governance of therapeutic products (e.g., natural health products, herbs) and medical devices (e.g., acupuncture needles) are also beyond this study’s scope.

We define *government reimbursement* as any type of government policy or program that explicitly pays for, subsidizes or reimburses, and/or requires or permits reimbursement or payment for the therapeutic services of unconventional (T&CM) practitioners.

### Source selection & data collection

The present study draws primarily on document-based data. We began our work by identifying the range of unconventional (T&CM) therapeutic practices subject to statutory provisions across the US. We did so primarily with reference to prior document-based compilations of relevant data (i.e., secondary sources). These documents (all of which are cited in the Background section of this work) included: scholarly publications; international and national reports related to T&CM governance; websites and grey literature reports published by US government bodies, T&CM focused organizations, and T&CM practitioner associations. Having identified a shortlist of practitioner and practice types governed by various statutory provisions, we referred to the aforementioned sources to identify preliminary data pertaining to each of the following points, by jurisdiction: type/mechanisms of governance (e.g., professional licensure or other); year the relevant regulations were introduced; restricted/protected titles granted; legal scopes of practice; professional entry requirements; accreditation of training programs; and, forms and extent of government reimbursement. Using an Excel spreadsheet, we drew upon the identified documents to record information pertaining to each of the aforementioned points. To ensure accuracy and fill in gaps, we sought (wherever possible) to confirm each data point derived from a secondary non-governmental source (e.g., grey or scholarly literature) with reference to a primary, governmental or other legal source (e.g., law/statute, policy, rules and regulations, court ruling). Overall, we constitute primary rather than secondary sources as authoritative. For each recorded data point, we documented the source(s) used. To help characterize the landscape of T&CM practitioner governance across the US, we subsequently referred to our existing set of secondary sources, and other primary sources (e.g., peer-reviewed research, US government reporting) to compile available data, for statutorily-governed occupations, regarding: a) the number of practitioners nationwide; b) the occupation’s gender profile; and c) practitioners’ average annual income.

### Analysis

This work primarily uses a descriptive content analytic approach to interpreting the collected data [121]. Our analytic aim is to present a detailed but accessible summary of T&CM practitioner governance across the US, drawing attention to commonalities and differences across jurisdictions and practitioner types. Following collection of sought data jurisdiction by jurisdiction, we collated these data, by occupation and practice type, assembling a descriptive account that includes both quantitative ‘counts’ of the number of jurisdictions where particular governance approaches are evident, and a qualitative/narrative account of the governance approaches themselves.

### Presentation of results and citation approach

The Results section that follows synthetically presents the study findings in three ways: a) through maps; b) using tables; and c) in narrative form. Where scholarly sources are the sole or primary source for particular data included in the Results section, we provide explicit attributions. However, in alignment with similar policy-summative works (e.g., [18, 19, 92, 96, 122]), we do not directly cite (the hundreds of) state laws/statutes, and/or related rules and court rulings individually sourced for this work, though the reader may understand these to represent the primary source of study data. Nor do we cite, within the Results section, the grey literature sources that represent prior ‘compilations’ of regulatory (or related) data for particular T&CM practitioner groups and/or practices, which we used as a starting point for many lines of investigation. However, readers may refer to the Introduction section of this work, where we cite all of these grey literature sources. Readers will observe that to address the persistent problem of ‘dead’ and ‘disappearing’ online links for grey literature sources without a digital object identifier (DOI), we provide references to recent ‘snapshots’ of such sources from the Internet Archive Wayback Machine (archive.org), rather than direct links to the original sources we accessed. In cases where these documents were not already stored on the Wayback Machine, we have added them there to facilitate production of reliable citations for this work.

We now turn to the study results.

## Results

**Fig 2:**
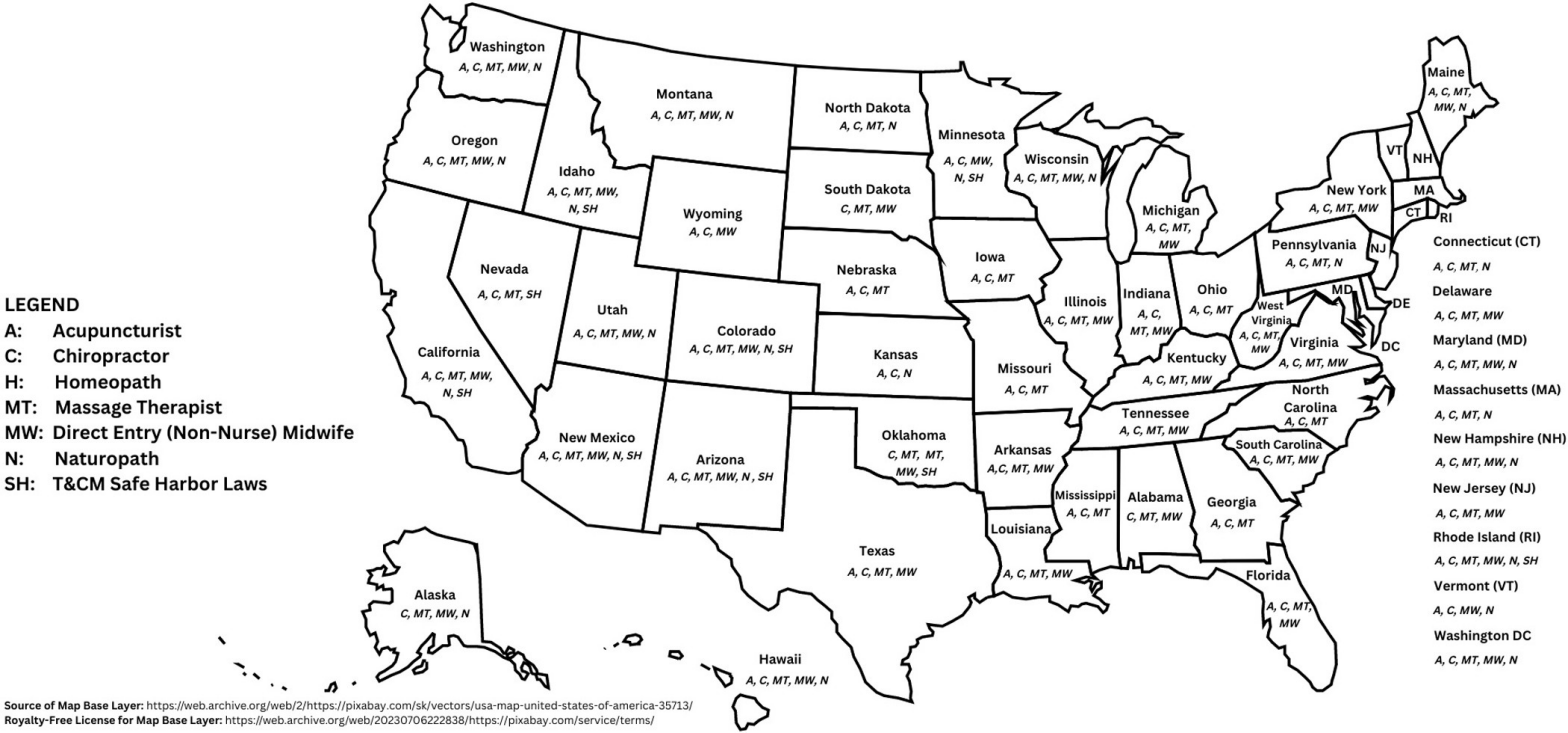
Statutory regulation of traditional and complementary medicine practitioners in the United States.

**Fig 3:**
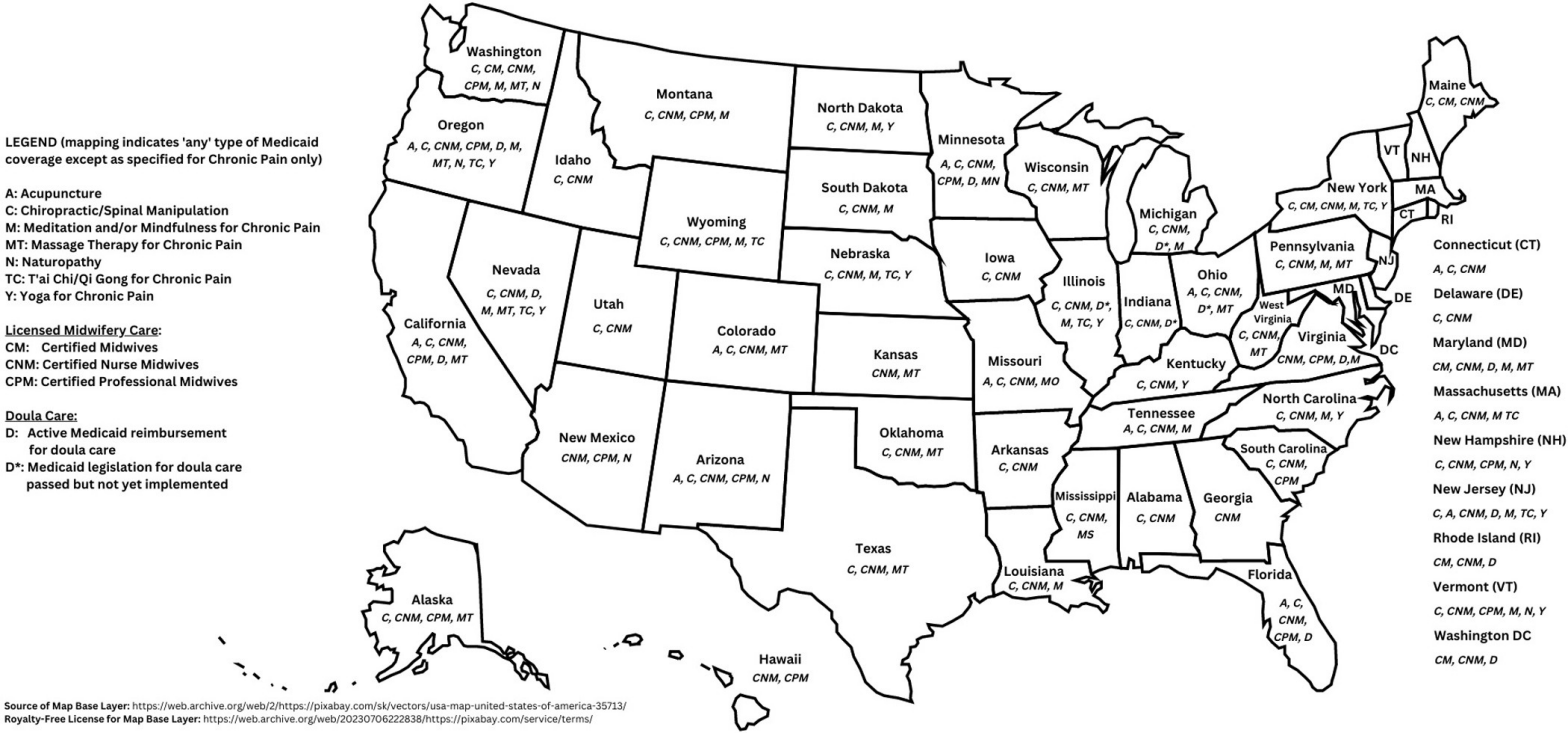
State medicaid coverage for traditional and complementary medicine practices and/or practitioners in the United States.

In what follows, we provide an overview of the T&CM occupations in the US that are subject to some form of statutory governance, as well as information about government-funded reimbursement for such services, where such exists. Readers may refer to Table 1 for a synthetic overview of these findings, along with additional details about T&CM practitioner governance and reimbursement by occupation and by jurisdiction. Table 2 provides an overview of training requirements for the five most widely-licensed T&CM occupational groups (acupuncturists, midwives, chiropractors, massage therapists and naturopaths); and Table 3 details the demographic features of these same five professions. Fig 2 provides a graphical representation of T&CM professional licensure (and ‘negative’ licensing laws) across the country. Fig 3 graphically represents, in binary (yes/no) form, the availability of any form of state Medicaid coverage for T&CM practices and/or practitioners across the US. Please note that all figures represent author compilations of data from a range of sources; however, Fig 2 draws substantially on the work published by McKay and colleagues with respect to government-funded insurance coverage for the non-pharmacological treatment of chronic pain [92].

### Acupuncturists

#### Governance, protected titles and scope

*Licensed acupuncturists*. Acupuncture practitioners trained in East Asian traditional approaches are licensed almost universally across the US, in forty-seven states, DC and four territories. The most commonly-used statutory title for traditional acupuncturists in the US is Licensed Acupuncturist (LAc). However, a few jurisdictions use alternate or additional titles; for example, Registered Acupuncturist (RAc) in the state of Colorado, Acupuncture Physician (AP) in Florida, and Doctor of Acupuncture (DAc, DAcu) in Hawaii, Rhode Island and Florida. In thirty states, the practice of Chinese herbal medicine is included in the statutory scope of practice of Licensed Acupuncturists.

*Auricular acu-technicians*. Twenty-four states have introduced statutory provisions to permit persons otherwise not licensed in acupuncture to practice auricular (ear) acupuncture, also termed the ’5-needle protocol’ (5NP), to treat persons with mental health conditions, addictions, pain and trauma. Statutory provisions in fourteen of these states permit lay persons with defined training to perform the 5NP, usually under the supervision of a licensed acupuncturist or a medical doctor. Nine states permit auricular acupuncture provision by certain health care professionals (e.g., nurses, physician assistants, occupational therapists, emergency medical services professionals, correctional medical providers), mental health care professionals (e.g., psychologists, counsellors, therapists), and/or social service professionals (e.g., social workers). Some jurisdictions have implemented restricted titles for exclusive use by auricular acupuncture practitioners certified, registered or licensed by the state; these titles include Licensed Auricular Detoxification Specialist and Licensed Auricular Detoxification Technician.

**Table 1:** Statutory governance and reimbursement profile of traditional and complementary medicine occupations in the United States.

T&CM Occupation	Statutory Regulation (Licensure (Yes / No)	Earliest / Latest Laws	US Jurisdictions with Regulations Explicitly Governing T&CM Practitioners		Insurance Reimbursement (Public / Private; n = # of jurisdictions)
			*#*	*States*, *Territories*, *District of Columbia (DC)*	
***Acupuncturist***	Y: *Acupuncturist* *(East Asian medicine practitioner)*	1973 / 2017	52	AK, AZ, AR^h^, CA^h^, CO^h^, CT^h^, DE^h^, DC^*^, FL^h^, GA^h^, HI^h^, ID ^h^, IL^h*^, IN, IA, KS^h^, KY, LA, ME, MD, MA^h*^, MI, MN^h^, MS^h^, MO, MT^h^, NC^h^, ND^h^, NE, NH, NJ^h*^, NM^h^, NV^h^, NY^h^, OH^h*^, OR^h^, PA^h*^, RI^h^, SC, TN, TX^h^, UT, VT^h*^, VA, WA^h^, WV^h^, WI^h^, WY, AS, GU, MP, PR ^h^ States that include Chinese herbology in the scope of practice for Acupuncturists (n = 30) *Additional certification required to practice Chinese herbal medicine (n = 7)	**Public:** Medicare (reimburses acupuncture as a modality but acupuncturists may not bill directly; Veterans Health Administration; Medicaid (n = 6): CA, MA, MN, NJ, OH, OR **Private:** All jurisdictions
	Y: *Auricular* *Acu-Technician*	1989 / 2021	24	‘Anyone’(n = 14): AR,^1^ AZ,^1^ CT,^2^ GA,^1/2^ IN,^1/2^ LA,^1/2^ MI,^2^ MO,^1^ MS,^1^ NM,^1^ NY,^1^ SC,^1/2^ TN,^1^ VA^1/2^ Licensed health / mental health care professionals and/or social service practitioners only (n = 9): MD,^1/2^ ME,^1^ NH,^1/2^ RI,^1^ TX,^1/2^ CO,^3^ DE,^3^ UT,^3^ WV^3^ ^1^*With supervision of*: ^*1*^*Licensed acupuncturist*, *and/or* ^2^*MD/DO;* ^3^*Without required supervision*.	None identified
	Scope inclusion	—	—	Acupuncture and/or dry needling included in scope of various licensed health professionals; see Table 2.	—
***Birth Worker***	Y: *Nurse-Midwife*		all	Certified Nurse Midwives (CNM) universally regulated across all US jurisdictions	**Public:** Medicare, Tricare, Medicaid (mandated in all states) **Private:** All jurisdictions
	Y: *Direct-Entry Midwife*	1976 / 2021	36	AL, AK, AZ, CA, CO, *DC, *DE, FL, *HI, ID, IL, IN, KY, LA, MD, *ME, MI, MN, MT, NH, *NJ, NM, *NY, *OK, OR, *RI, SC, SD, TN, TX, UT, VT, *VA, WA, WI, WY *Jurisdictions recognizing ‘Certified Midwife’ (CM) credential (n = 10) in addition to ‘Certified Professional Midwife’ (CPM) designation (n = 36)	**Public**: Medicaid for CPM (n = 13): AK, AZ, CA, FL, MN, MT, NH, NM, SC, WA, VA, VT, WY Medicaid for CM (n = 6): DC, ME, MD, NY, RI, WA **Private**: All jurisdictions for CM; much more limited coverage for CPMs
	Scope inclusion: *Naturopathic Doctor*		6	CA, MT, NH, OR, UT, VT	See *Naturopaths*, below
	N: *Birth Doula*	2011 / 2022	17	Medicaid reimbursement(n = 10): CA, FL, MD, MN NJ, NV, OR, RI, VI, DC Medicaid reimbursement legislation passed but not fully implemented (n = 4): IL, IN, OH, MI Voluntary state doula registries (n = 3): AZ, LA, WA Hospital privileges (n = 1): CO	**Public:** Medicaid (n = 10) **Private:** Very few plans cover doula care
***Chiropractor***	Y: *Chiropractic Doctor*	1913 / 1974	all	Chiropractors universally licensed across all US jurisdictions	**Public**: Medicare (Chiropractors may bill Medicare directly); Veterans Health Administration; Medicaid (n = 43): all except DC, GA, HI, KS, MD, NM, RI, VA **Private**: All jurisdictions
	Y & N: *Chiropractic Assistant / Technician*	∼1970 / 2022	30	Licensed (n = 4): ME, MD, NJ, *WA Unlicensed but recognized in statute (n = 26): AK, AR, AZ, CA, CO, DC, FL, GA, IA, ID, *LA, MA, MS, NC, NV, NM, ND, OH, OK, OR, SD, TN, TX, UT, WV, WY *Chiropractic *X-Ray* Technicians exclusively mentioned in statute	Not reimbursed independently of Chiropractic Doctors.
***Homeopath***	Y: *Homeopathic Physician (MD/DO)*	1988 / 2009	3	AZ, CT, NV	None identified
	Y: *Homeopathic Assistant*	1988 / 2008	2	AZ, NV	None identified
***Massage Therapist / Bodyworker***	Y: *Massage Therapist* *(‘Western’ massage [W]) and Asian Bodywork Therapist [ABT])*	1915 / 2021	50 [W] 10 [ABT]	AL, AK, AZ, AR, CA, CO, CT, *DC, DE, FL, GA, HI, ID, IL, IN, IA, KS, KY, *LA, ME, *MD, MA, MI, *MS, *MO, MT, NE, NV, **NH, *NJ, NM, NY, *NC, ND, OH, OR, PA, *RI, SC, SD, TN, TX, UT, VT, VA, WA, WV, *WI, PR, VI *In addition to recognizing practitioners of ‘Western’ massage therapy, *these states also offer massage therapy licensure to practitioners of ‘Asian Bodywork Therapy’ (ABT)*. **NH licenses ABT as a stand-alone profession (in addition to reflexology and structural integration).	**Public:** Medicare Advantage (pain only, n∼270); Veterans Health Administration (18 Whole Health sites only); Medicaid: some waiver & managed care plans. **Private:** Workers’ Compensation & Personal Injury Protection (most); few private health plans
	Y: *Reflexologist*		5	ND, NH, NV, TN, WA	None identified
	Y: *Structural Integrator*		2	ND, NH	None identified
***Naturopath***	*Y*: *Naturopathic Doctor*	1919 / 2022	26	AK, AZ, CA, CO, CT, DC, HI, ID, KS, ME, MD, MA, MN, MT, NH, NM, ND, OR, PA, RI, UT, VT, WA, WI, PR, VI	**Public:** Medicaid (n = 6): AZ (minors only), NM, NH, OR, WA, VT **Private**: AZ, AK, CO, CT, HI, ME, MT, NH, OR, UT, VT, WA
	*N*: *Naturopathic Assistant*	2011	1	CA	None identified
	*N*: *Colon Hydrotherapist*	2022	1	WA	None identified
***Psychedelic Facilitator***	*N*: *Religious Exemption*	1978 / 2009	all 1	Native American Church: Exemption (Federal Law) for ceremonial use of peyote Uniao do Vegetal Church: Exemption (Supreme Court ruling) for ceremonial use of ayahuasca Church of Santo Daime: Exemption (Oregon State Court ruling) for ceremonial use of ayahuasca in Oregon state only	None identified
	*Y*: *Psychedelic Facilitator*	2022	1	OR	None identified
***Safe Harbor Laws for T&CM Practitioners***	N: *Unlicensed T&CM Practitioners*	1976 / 2015	10	AZ, CA, CO, ID, LA, MN, NM, NV, OK, RI	None identified

Source: Author compilation.

**Table 2:** Training requirements for major licensed T&CM professions in the US.

T&CM Profession	Educational Requirements
***Acupuncturist***	1900+ hours
***Midwife***	Graduate degree OR 3-year, full-time training OR portfolio evaluation
***Chiropractor***	4200+ hours
***Massage Therapist***	500+ hours
***Naturopath***	4100+ hours

Source: Author compilation.

**Table 3:** Demographic features of major licensed T&CM professions in the US.

T&CM Profession	# of Licensed Practitioners	% Female	Mean Annual Income
***Acupuncturist***	28,000	71.3%	$71,770 (Mean)
***Midwife***	13,600 (Nurse-Midwives) 2600 (Direct-Entry Midwives)	Unknown (Nurse-Midwives) 98.9% (Direct-Entry Midwives)	$114,210 (Mean; Nurse-Midwives) Unavailable (Direct-Entry Midwives)
***Chiropractor***	77,000	31.8%	$81,240 (Mean)
***Massage Therapist***	334,000	86%	$49,260 (Mean)
***Naturopath***	6000	74%	Unavailable

Source: Author compilation; Income data (year: 2021) from US Bureau of Labor Statistics [123–126].

**Fig 4:**
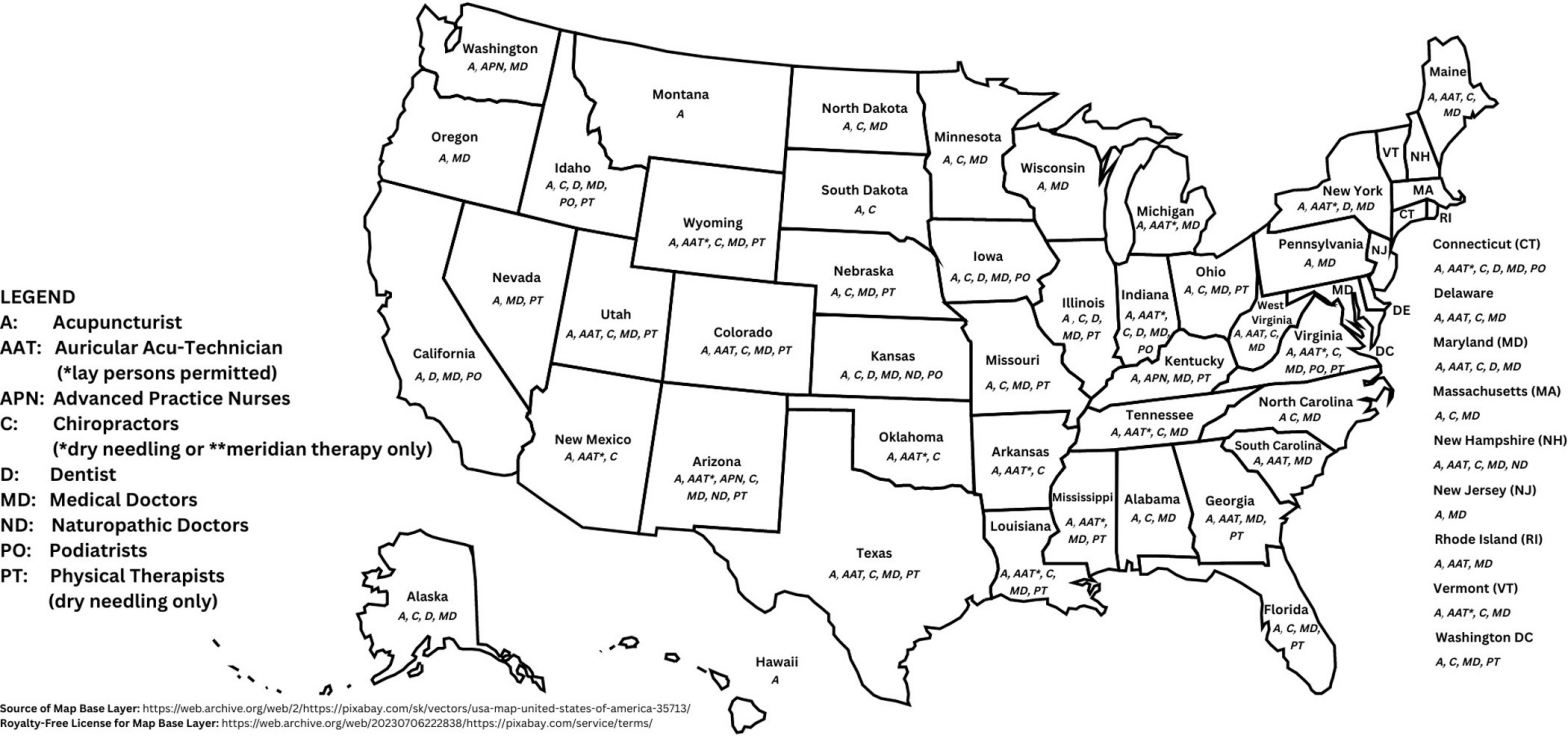
Legal practice of acupuncture & related techniques (e.g., Dry needling) in the United States.

*Non-acupuncturist health care professionals*. As shown in Table 4 and on Fig 4, several licensed health care occupations are permitted, in some states, to practice acupuncture (or similar techniques involving the bodily insertion of filiform needles, such as dry needling and ’meridian therapy’) more broadly within their scopes of practice. No additional protected titles are known to be implemented in these cases.

**Table 4:** US jurisdictions legally sanctioning the use of acupuncture (or similar practices) in the scope of non-acupuncturist health care professionals.

Profession	# of States / Territories (*practice authorized*)	Required Training	Jurisdictions
***Chiropractor***	36 *(acupuncture*, *except as specified*)	Unspecified	CT, KS, MD (dry needling only), MA and NH (dry needling only), NM (meridian therapy only), MA
		≤100 h	AL, AZ, AR, CO, DE, FL, IL, IA, LA (dry needling), MN, MO, NE, ND, OK, SD, TX, UT, WV, WY
		200–250 h	DC, ID, IN, ME, NC, TN, VA
		300 h	AK (dry needling only), OH, VT
		200–250 h	DC, MD, MS, PA, VA
		300 h	GA, LA, MI, NJ, NY, RI, SC
***Dentist***	10 *(acupuncture)*	Unspecified	AK, CT, IL, IA, KS, MD
		≤100 h	CA
		200 h	IN
		300 h	ID, NY
***Medical Doctor***	44 *(acupuncture)*	Unspecified	AK, AL, AZ, CA, CO, CT, DE, FL, IA, ID, IL, IN, KS, KY, ME, MA, MN, MO, NC, ND, NE, NV, NH, OH, OR, TN, TX, UT, VT, WA, WV, WI, WY
		200–250 h	DC, MD, MS, PA, VA
		300 h	GA, LA, MI, NJ, NY, RI, SC
***Naturopathic Doctor***	3 *(acupuncture)*	250 h	AZ
		500 h+	KS, NH
***Nurse***	3 *(acupuncture)*	Unspecified	Acupuncture in scope of ‘Advanced Practice Nurses’ only: AZ, KY, WA
***Physical Therapist***	38 *(dry needling only)*	Unspecified	AZ, DC, KY, MO, NE, OH, TX
		≤100 h	CO, FL, GA, ID, IL, LA, MS, TN, VA, WY
		150 h	NV
		300 h	UT
***Podiatrist***	7 (acupuncture)	Unspecified	CT, IA, KS
		≤100 h	CA
		200 h	IN, VA
		300 h	ID

Sources: Authors’ compilation from primary sources, informed by [19, 20]

#### Professional entry requirements

*Licensed acupuncturists*. While training requirements vary somewhat by state, licensed acupuncturists across the US generally complete a minimum of 1900 hours of related training, along with a professional entry exam. Candidates for acupuncturist licensure in all jurisdictions except California are required to complete at least three of four national certification examinations delivered by National Certification Commission for Acupuncture and Oriental Medicine (NCCAOM). These examinations, which are available in the English, Chinese and Korean languages, address: a) East Asian medical theory; b) Biomedical sciences; c) Acupuncture and point location; and d) Chinese herbal medicine. Across the thirty states that include Chinese herbal medicine within the licensed acupuncture profession’s scope of practice, twelve require completion of the NCCAOM’s Chinese herbology exam in order to practice. The state of California’s Acupuncture Board administers an independent licensing examination—offered in English, Mandarin (Chinese) and Korean languages—to candidates in that jurisdiction; Chinese herbal medicine is included within that state’s acupuncturist scope.

*Auricular detoxification practitioners*. Training requirements for auricular detoxification practitioners vary by state, but generally require completion of trainings lasting at least three full days; some jurisdictions require certification with particular organizations (e.g., NADA), and some explicitly require additional completion of clean needle training.

*Non-acupuncturist health care professionals*. As shown in Table 4, statutory training requirements for non-acupuncturist, licensed health care professionals who are permitted to use acupuncture needles in their clinical scope vary considerably by profession and by jurisdiction.

**Reimbursement.** Medicare-eligible persons with chronic low back pain may be reimbursed for 12 acupuncture sessions over a 90-day period, which may be extended to a maximum of 20 treatments over twelve months if improvement is evident. However, it is important to note that licensed acupuncturists may not directly bill Medicare. As such, the care of licensed acupuncturists may only be reimbursed by Medicare if performed under the direct supervision of a billing provider (e.g., MD/DO, nurse practitioner); further, the reimbursement rates vary between different types of providers. Acupuncture services for pain and other conditions (by licensed acupuncturists, medical doctors and chiropractors) are widely available at Veterans Affairs Medical Centers and sometimes also reimbursed for veterans receiving care at other health care facilities. In addition, auricular acupuncture treatments (termed ‘battlefield acupuncture’ in this context) are made available at many Veterans Affairs facilities and, depending on local state laws, may be additionally delivered by health professionals other than those listed above. While Tricare does not generally cover acupuncture, some major Tricare facilities, such as the Integrative Health and Wellness service at the Walter Reed National Military Medical Center, and the Acupuncture and Integrative Medicine Center at Joint Base Andrews, do include acupuncture (for a variety of conditions) in their reimbursed offerings. In addition, ongoing acupuncture and auricular acupuncture training for medical professionals are offered at Walter Reed.

### Birth workers (Midwives & doulas)

**Fig 5:**
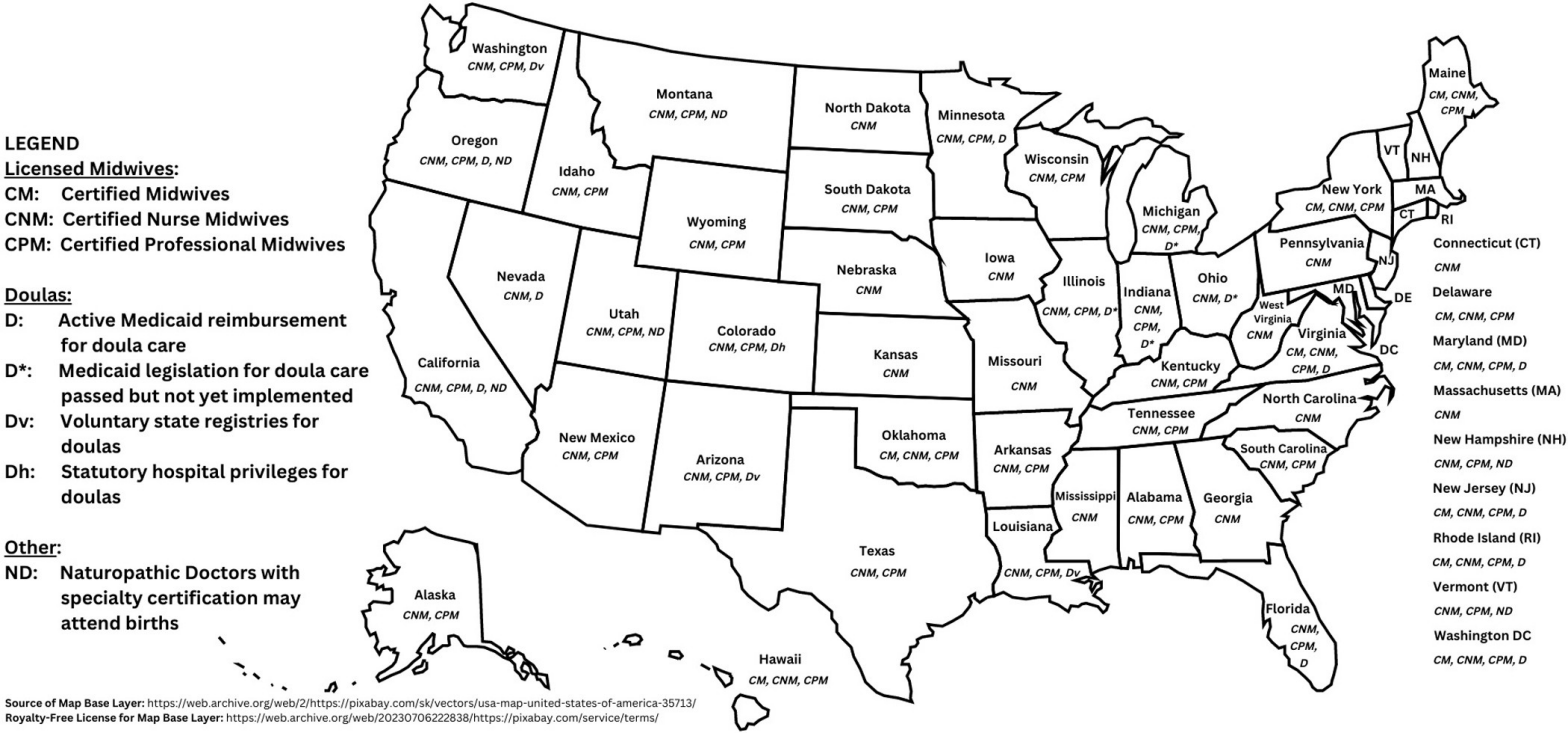
Statutory recognition of non-obstetrician birth workers in the United States.

Fig 5 provides a graphical overview of the statutory governance of non-obstetrician birth workers in the US, as elaborated in what follows.

#### Governance, protected titles and scope

*Midwives*. There are three distinct pathways to midwifery licensure in the US, each with its own protected titles, scope of practice and professional entry requirements. The first group of statutorily regulated midwives, who are universally licensed across all states and territories and DC, also hold a Registered Nurse license. They typically carry the protected title of Certified Nurse-Midwife / Certified Registered Nurse Midwife (CNM) or, in some jurisdictions, the titles of Licensed Nurse-Midwife (LNM) or Nurse Midwife. The second and third groups of midwives, together characterized as ‘direct-entry’ midwives, do not carry nursing credentials; and are often identified using the protected titles of Licensed Midwife (LM) Registered Midwife (RM) in the jurisdictions where they are regulated. One group of direct-entry midwives, who typically earn a ‘Certified Midwife’ credential, are licensed across nine states and DC; the other group, referred to as Certified Professional Midwives (CPM), are licensed in 35 states and DC.

All three types of licensed midwives have similar scopes of practice with respect to supporting pregnancy, childbirth and postpartum care. However, CNMs and CMs additionally have hospital-attending privileges, as well as statutory authority to provide comprehensive health care across the lifespan, including diagnosis and treatment (e.g., medication prescribing). While CPMs do not generally have medication-prescribing authority, they may access and administer certain otherwise-restricted medications in some states; CPMs are also the only group of the three trained to attend births outside of hospital settings.

Across the US, there remain midwives—usually referred to as traditional midwives—who for reasons personal, religious or philosophical, may elect not to become formally credentialed or licensed. Such midwives risk criminal prosecution for practicing nursing or medicine without a license, as do CMs and CPMs in jurisdictions where direct-entry midwives are not licensed.

*Naturopaths*. In six of the 26 states with naturopathic medical licensure laws (elaborated further on), naturopathic doctors who have completed additional training in ‘naturopathic obstetrics’ may attend childbirths (much like midwives) within the scope of their naturopathic professional license.

*Birth doulas*. Birth doulas are not licensed anywhere in the US, but are increasingly recognized in statute, generally with the aim of providing Medicaid reimbursement for doula services. In nine states and DC, Medicaid programs are actively reimbursing doula care following related legislation. The regulatory landscape in this area is rapidly changing, with new developments emerging at the time of this work’s publication. Four states have for example passed legislation to reimburse doula care but implementation is not yet complete. As elaborated in the section on reimbursement below, additional related reimbursement programs (not formally entrenched in statute) are in place in other states as well.

Three states (Arizona, Louisiana, and Washington) have also introduced voluntary state registries for birth doulas with the aim of exploring Medicaid reimbursement in future; and Colorado has uniquely implemented a statutory provision that permit doulas (as non-medical personnel) to attend hospital births where only family members were previously permitted to be present.

#### Professional entry requirements

*Midwives*. Educational pathways differ considerably for the three statutorily-recognized categories of midwives in the US. Certified Nurse-Midwives are uniquely required to hold a Registered Nurse license in addition to their midwifery credential. Aside from this requirement, however, pathways to licensure for Certified Nurse-Midwives and Certified Midwives are similar. Licensure candidates in both groups must complete a graduate-level degree program recognized by the Accreditation Commission for Midwifery Education (ACME) (lasting a minimum of 24 months), and a licensing examination administered by the American Midwifery Certification Board.

There are, by contrast, multiple routes to candidacy for the Certified Professional Midwife credential, which is widely-recognized by regulators in states where direct-entry midwives are licensed. Formal training options include completion of: a) a graduate-level degree program recognized by ACME (as above), along with documentation of at least ten non-hospital based birth experiences; or, b) a midwifery education program (usually lasting at least three years) recognized the Midwifery Education Accreditation Council. Another option, in particular for experienced, apprenticeship-trained and internationally-trained midwives, is to successfully complete a portfolio evaluation process through the North American Registry of Midwives (NARM). All certification candidates (both formally-trained and portfolio-validated) must subsequently complete a certification examination administered by NARM.

*Naturopaths*. Naturopathic Doctors licensed in the six states whose practice scope stipulations include naturopathic childbirth attendance as an optional certification are required to complete additional training, along with an examination administered by the American College of Naturopathic Obstetricians. Educational requirements stipulated (beyond standard naturopathic training) by individual states range from 100 (California, Montana, New Hampshire, Utah) to 200 (Oregon, Vermont). These didactic requirements are typically paired with a requirement to attend a minimum number of childbirths, which vary from 15 (Utah, California) to 50 (Montana, Oregon, Vermont).

*Birth doulas*. Most jurisdictions where birth doula services are eligible for Medicaid coverage have implemented voluntary state registries where birth doulas seeking reimbursement privileges may be listed. Inclusion on a state registry is generally contingent on a birth doula’s adherence to state-specified training standards; some states (e.g., in Maryland, Minnesota) require instead that Medicaid-eligible doulas be certified with external (i.e., non-governmental) organizations such as (e.g., DONA). Generally speaking, training requirements for Medicaid-eligible birth doulas are in the 16-hour range, while some states require more extended training (e.g., Oregon, 40 hours). Some states (e.g., California) also offer an ‘experience pathway’ to Medicaid-eligibility, contingent on prior documented work as a birth doula (rather than formal training).

#### Reimbursement

*Midwives*. Third party reimbursement for midwifery varies considerably by licensure type, with care provided by Certified Nurse-Midwives being most extensively covered. Certified-Nurse Midwives are eligible for Medicare and Tricare reimbursement federally, a privilege not granted to licensed, direct-entry midwives. Medicaid programs in all US states similarly reimburse care delivered by Certified Nurse Midwives; however, Medicaid coverage extends to Certified Midwives and Certified Professional Midwives in just five and thirteen states, respectively. While most private insurance plans across the US reimburse care provided by Certified Nurse-Midwives and Certified Midwives, only six states mandate private coverage for Certified Professional Midwives.

*Birth doulas*. As noted earlier on, Medicaid coverage for the work of birth doulas is ongoing in nine states and DC, with implementation in process in four more states. In three more states (Kentucky, Missouri and Texas), Medicaid coverage for doula care is not entrenched in statute, but some managed care organizations nonetheless offer reimbursement to Medicaid recipients who receive doula services. In New York City, a municipal initiative is in place to reimburse doula services for Medicaid recipients, but no related legislation is in place in New York state as a whole. Seven states (Delaware, Georgia, Iowa, Massachusetts, Oklahoma, Tennessee, Texas), and the federal Tricare program, have pilot programs or research studies presently underway to explore reimbursement for doula care.

### Chiropractors

#### Governance, protected titles and scope

Chiropractors are regulated across all 50 US states, the District of Columbia and five territories. Across these jurisdictions, chiropractors are authorized to use the protected title of Doctor of Chiropractic (DC). Chiropractic scopes of practice vary by jurisdiction, with spinal manipulation, physical examinations, and the use of diagnostic imaging (e.g., x-ray, CT, MRI, ultrasound), blood analysis, lifestyle counseling, nutrient supplementation being almost universally included. Many jurisdictions also include additional diagnostic procedures (urinalysis, fecal analysis, throat swab, pre-employment physicals, breast and genital exams) and treatment options (e.g., magnetic therapy, short wave therapy, massage, homeopathy, colonic irrigation) within the scope of chiropractic practice. Chiropractors are permitted to practice acupuncture (and/or related practices such as dry needling) is permitted in about two-thirds of jurisdictions. A wide range of additional diagnostic and treatment approaches are included in chiropractors’ scope in a minority of jurisdictions (e.g., hypnosis, electrolysis, minor surgery, limited drug prescribing, vitamin injection). In six states, chiropractors are recognized as primary care providers.

Chiropractic Assistants are recognized in statute in thirty jurisdictions. While the parameters of the Chiropractic Assistant role vary by state, commonly-delegated tasks include patient history taking and administration of low-risk therapeutic procedures. Three states license Chiropractic Assistants; Washington state, an outlier, licenses Chiropractic X-Ray Technicians as a standalone profession. Elsewhere, state ‘certification’ or ‘registration’ is granted to Chiropractic Assistants who meet professional entry requirements. The state of Louisiana uniquely uses a state certification mechanism to govern Chiropractic Assistants with an exclusive focus on the conduct of chiropractic x-rays.

#### Professional entry requirements

Educational requirements for professional licensure of chiropractors are uniform across the US, consisting of: a) Four to five years (4200 hours) of formal training at one of twenty educational institutions accredited by the Council of Chiropractic Education; and, b) a professional entry examination delivered by the National Board of Chiropractic Examiners. Licensure and state certification/registration requirements for Chiropractic Assistant vary across the country. Many jurisdictions require that Chiropractic Assistants meet minimum, jurisdiction-specific training requirements (e.g., Mississippi, Oklahoma, Tennessee, South Dakota, North Carolina, Oregon). Other states (e.g., Idaho, Ohio) statutorily recognize Chiropractic Assistants but stipulate no minimum training requirements.

#### Reimbursement

Some aspects of chiropractic care are eligible for reimbursement through the federal Medicare and VA insurance programs; and in 41 states, through Medicaid as well. While Chiropractors (who are considered physicians under Medicare) may bill Medicare directly for their services, chiropractic services eligible for Medicare coverage are limited to spinal manipulation. By contrast, veterans may access a range of chiropractic services—including spinal manipulation, manual therapies, acupuncture, and other non-pharmacologic approaches—at VA health facilities and VA-affiliated community care settings. While chiropractic care is not reimbursed by Tricare, active-duty service members and activated national guard/reserve members of the US military may access chiropractic care by referral at specific military hospitals or clinics. Across the 43 states where chiropractic care is eligible for Medicaid coverage, benefits vary: ranging from eight to fifty treatments per year, and/or at times limited to a specific dollar amount (e.g., Mississippi, $700). Chiropractic care is also widely reimbursed by private insurance across the United States.

### Homeopaths

#### Governance, protected titles and scope

Homeopathy is not regulated as a standalone profession in any US jurisdiction. However, homeopathic practice is recognized in statute in several states. In jurisdictions where naturopathy is licensed, homeopathy is included within that profession’s scope; a few states also include homeopathy within the statutory scope of licensed acupuncturists, chiropractors and other regulated professionals. Furthermore, three states (Arizona, Connecticut and Nevada) license medical doctors (MDs and DOs) who wish to specialize in homeopathy using the protected title of ‘Homeopathic Physician’. Arizona and Nevada furthermore provide statutory certification/registration to non-physician ‘Homeopathic Assistants’ working under the supervision of a Homeopathic Physician.

#### Professional entry requirements

Medical doctors (MDs/ DOs) seeking licensure as Homeopathic Physicians in their states must fulfill postgraduate training requirements in homeopathy in addition to being in good standing with their state medical boards. These requirements vary from state to state; and range from 120 hours (Connecticut) to 600 hours (Nevada), with some states additionally requiring a licensing examination. Homeopathic Assistants training requirements range from 200 to 400 hours.

### Massage Therapists / Bodyworkers

#### Governance, protected titles and scope

Massage therapy is statutorily regulated almost universally across the United States, in 47 states, DC, and two territories (Puerto Rico, US Virgin Islands). Regulation across these jurisdictions is primarily implemented via licensure regimes, except in the state of California, where massage therapists meeting defined entry requirement may voluntarily certify on a state registry. Currently, Kansas, Minnesota and Wyoming are the only US jurisdictions that do not statutorily govern massage therapy practitioners. Forty-four US jurisdictions protect the title of Licensed Massage Therapist (LMT) in statute for the profession’s exclusive use; other, less frequently used protected titles include CMT (Certified Massage Therapist), RMT (Registered Massage Therapist) and MMT (Master Massage Therapist). In addition to restricting these titles, some state regulations restrict use of the term ‘massage’ in the context of a paid service for exclusive use by licensed massage therapists.

Statutory definitions of massage therapy vary across jurisdictions. Many states constitute licensed massage therapy as a structured system of touch to the soft tissues; such touch is often characterized as having the purpose of relaxation, promotion of well-being, along with therapeutic effects. While many statutes characterize massage therapy as a health care service / treatment, licensed massage therapists do not have statutory authority to diagnose or treat disease. However, in several states, statutes indicate that licensed massage therapists may ’treat dysfunction’, ’provide pain relief’ or ’enhance tissue function’ based on an ’assessment’ of the client’s bodily condition. Many state regulations demarcate a set of specific practices as falling within the boundaries of licensed massage therapy; these commonly include manual therapy techniques (e.g., pressure, compression, kneading, stretching), the application of heat or cold, and topical applications.

Several states also make explicit which ’styles’ or ’systems’ of bodywork (and at times, other practices) are or are not included within the profession’s statutory scope. Alaska, for example, delineates the massage therapist profession as exclusively governing the practice of ’traditional European or contemporary western massage therapy’, while explicitly excluding the bodywork practices of structural integration (’Rolfing’), reflexology, and the ’traditional practices of Native American traditional healers’. Hawaii uniquely includes Lomilomi (an Indigenous Hawaiian form of bodywork) within massage therapists’ statutory scope. In at least nine states, practitioners of several systems of Asian Bodywork Therapy may be licensed under state massage therapy boards along with practitioners of ‘Western’ styles massage therapy. In Washington DC, the state statute includes an extended list of bodywork styles that fall under massage therapy’s auspices (including but not limited to "Rolfing, Neuromuscular Therapy, Shiatsu or acupressure, Trigger Point massage, Trager, Tui na, Reflexology, Thai Massage, deep tissue massage, Myofascial Release, Lymphatic Drainage, Craniosacral, Polarity, Reiki, Swedish Massage, and Therapeutic Touch”). Arizona’s statute, by contrast, simply indicates that all forms of ’bodywork therapy’ are constituted to fall under the profession’s scope, though other practices (e.g., colonic irrigation) are explicitly excluded. The state of Florida, conversely, explicitly includes colonic irrigation within massage therapists’ scope, for practitioners with additional certification in that field. Other jurisdictions (e.g., Colorado, Massachusetts, Indiana, Kentucky) do not explicitly define European/western massage therapy as being their implied reference point, but explicitly exclude from the profession’s purview a defined group of bodywork practices that vary by state, but include such practices as reflexology, Rolfing, polarity therapy, Feldenkrais, Trager, shiatsu, and reiki.

Five states license reflexology practitioners separately from massage therapists; two of these states also license practitioners of structural integration. In some jurisdictions (e.g., Nevada, Washington) reflexologist and/or structural integration licensure are implemented under separate units within the state massage therapy regulating board. Tennessee and North Dakota, by contrast, have distinct reflexology licensing bodies; and New Hampshire uniquely licenses reflexologists and two other types of bodywork practitioners under a standalone Board of Reflexologists, Structural Integrators and Asian Bodywork Therapists. Protected titles for reflexologists vary across these jurisdictions, and include: Licensed Reflexologist (North Dakota, Nevada), Registered Certified Reflexologist (Tennessee), and Certified Reflexologist (Washington); protected titles for practitioners of structural integration include: Licensed Structural Integrator (New Hampshire) and Licensed Structural Integration Practitioner (North Dakota). In some jurisdictions (e.g., Nevada, New Hampshire), state laws not only restrict (for licensees) the use of the aforementioned titles, but also the terms ‘reflexology’ and ‘structural integration’, and, notably, the practice of these therapeutic modalities. Elsewhere (e.g., Washington), however, these practices remain in the public domain, with licensure limiting the use of protected titles alone.

#### Professional entry requirements

While a national Federation of State Massage Therapy Boards supports state regulators “to ensure that the practice of massage therapy is provided to the public safely and competently,” professional entry requirements for licensed massage therapists are set by state regulators and vary across jurisdictions. All jurisdictions require at least 500 hours of training (usually in ‘Western’ massage therapy). Thirty-one jurisdictions have implemented minimum training requirements between 500 and 600 hours; nine require between 601 and 700 hours; three (list them) require 750 hours; and three (Nebraska, Puerto Rico, and New York state) require 1000 hours of massage therapy training. In addition, most jurisdictions require that candidates for massage therapy licensure complete one (of multiple) professional entry examinations, all of which require at least 500 hours of documented training but differ in key ways.

The MBLEx (Massage and Bodywork Licensing Examination), administered by the Federation of State Massage Therapy Boards, is an ‘entry level’ professional test accepted by regulators in 46 of the nation’s massage therapy-regulating jurisdictions; and is the only examination to be offered in both English and Spanish languages. A second examining agency, the National Certification Board for Therapeutic Massage and Bodywork, offers a ‘Board Certification’ examination which, like the MBLEx, is geared to candidates trained in ‘Western’ massage therapy, and is accepted interchangeably with the MBLEx by 40 jurisdictional regulators across the United States. Board certification by the NCBTMB is considered by some to be a more rigorous credential in Western massage therapy than MBLEx completion. In addition, nine state regulators accept (interchangeably with MBLEx and ‘Board Certification’) candidates trained in Asian Bodywork Therapy who successfully complete an examination which, until recently, was administered by the National Certification Commission for Acupuncture and Oriental Medicine (NCCAOM) but has been discontinued. Two jurisdictions (New York and Hawaii) administer state-specific massage therapy licensing examinations; and the state of California uniquely requires no professional entry exam.

While there is no single system of educational validation for massage therapy in the United States, multiple organizations implement accreditation mechanisms for massage therapy programs. The American Massage Therapy Association (AMTA) for example, welcomes ’member schools’ from across the country that meet a 500-hour minimum educational threshold. Another, smaller organization, the Alliance for Massage Therapy Education (AFMTE), similarly offers School Memberships to massage training programs of 500+ hours. The Commission on Massage Therapy Accreditation (COMTA) offers two forms of higher-level, voluntary recognition for massage therapy schools; the first endorses schools’ curricular competencies, and the second other more rigorously accredits programs as a whole. While neither AMTA, AFMTE or COMTA recognition is directly linked to massage therapist licensing, some (but not all) state massage therapy boards (and other governing bodies) use guidance from these organizations to endorse or validate particular educational programs where prospective licensees may train.

Statutory training requirements for licensed reflexologists across the aforementioned jurisdictions are generally in the 200-hour range, with some states (e.g., Nevada, North Dakota, Washington) additionally requiring a reflexology-specific licensure exam. Training requirements for licensed structural integration practitioners are generally above 700 hours.

#### Reimbursement

While massage therapy is not officially reimbursed by Medicare, up to 270 ‘Medicare Advantage’ plans—corporate programs contracted by the federal government to implement Medicare across the US—have been reported to reimburse pain-related massage therapy care. It is important to note that licensed massage therapists are not permitted to bill Medicare directly; and that other health care professionals (e.g., physical therapists) may also provide massage therapy services within their scope. Massage therapy is also available to veterans for various health conditions at the VA’s eighteen ‘Whole Health Flagship sites. With the exception of a few state Medicaid waiver plans, the services of licensed massage therapists are not generally reimbursed by Medicaid. However, some Medicaid ‘managed care’ plans, corporate programs contracted by state governments to implement Medicaid benefits, do include massage therapy in their coverage. Most Personal Injury Protection and Workers’ Compensation insurance payors reimburse for care delivered by licensed massage therapists. However, a minority of private health insurance payers reimburse licensed massage therapy care.

### Naturopathy

#### Governance, protected titles and scope

Naturopathic medicine is currently regulated across about half of US jurisdictions (23 states, DC and two territories). Across most of these jurisdictions (n = 24), licensed naturopaths are authorized the single protected title of Naturopathic Doctor (ND); in Arizona, both the ND and NMD (Naturopathic Medical Doctor) titles are statutorily protected; and in Idaho, only the NMD title is reserved for the licensed naturopaths’ usage. Nine states permit use of the term ‘physician’ by licensed naturopaths, a practice strictly prohibited in eight other jurisdictions.

Licensed naturopaths’ statutory scope varies by jurisdiction, with most permitting them to diagnose, prevent and treat disease via dietary and lifestyle counseling, the recommendation of nutritional supplementation and botanical medicines, use of physical medicine modalities including hydrotherapy, as well as homeopathy. In a limited number of states, licensed naturopaths are furthermore authorized to order laboratory testing (n = 23), blood draws (n = 18) and diagnostic imaging (n = 20), offer gynecological care (n = 17), provide intravenous therapies (n = 13), perform minor surgeries (n = 14), prescribe/administer a limited subset of pharmaceutical medications (n = 12) or other controlled substances (n = 9), attend childbirths (n = 6) and perform acupuncture (n = 3). Licensed naturopaths are recognized as primary care providers in twelve states.

In three states (Florida, South Carolina, Tennessee) the practice of naturopathy is illegal except by a medical doctor. In other states where naturopathic doctors are not currently licensed, it is often possible for naturopaths (including ‘traditional naturopaths’ trained outside of institutions accredited by the Council on Naturopathic Medical Education, described below) to practice in a more limited way as unlicensed health care practitioners.

Two states recognize additional categories of naturopathic personnel to support the work of naturopathic doctors. The state of California recognizes (but does not license) Naturopathic Assistants in statute, who may (under a licensed naturopath’s supervision): support patient intake and diagnostic screen (e.g., taking blood pressure and pulse readings, drawing blood and collecting other specimens for testing); administer medication through various means including via injection; and, bandage wounds. Washington state has a statutory certification mechanism for Colon Hydrotherapists working in a direct affiliation relationship with a state-licensed naturopath. No protected titles exist for either of these occupational groups.

#### Professional entry requirements

In co-ordination with the Federation of Naturopathic Medicine Regulatory Authorities, all jurisdictions that license naturopathic doctors across the US share uniform professional entry requirements, consisting of: a) four-years (at least 4100 hours) of training at one of four educational institutions accredited by the Council on Naturopathic Medical Education (CNME); and, b) the Naturopathic Physicians Licensing Examination, delivered by the North American Board of Naturopathic Examiners. All CNME-accredited naturopathic educational programs in the US are (along with their Canadian counterparts) members of the Association of Accredited Naturopathic Medical Colleges.

In California, Naturopathic Assistants must complete—under a licensed naturopath’s supervision—a minimum of 30 clock hours of specified training, as well as a specified number of injections and venipuncture/skin punctures in order to practice legally. Colon Hydrotherapists working under naturopathic supervision in the state of Washington must present credentialing in colon hydrotherapy with one of three external organizations (to a standard of approximately 265 hours), or complete equivalent direct training under the supervision of a licensed Washington naturopath, paired with a state board examination.

#### Reimbursement

While the care of licensed naturopathic doctors is not currently eligible for reimbursement by federal government health insurance programs, six state Medicaid programs do cover some aspects of naturopathic care. In Arizona, only pediatric naturopathic care is eligible for coverage. Furthermore, some degree of private insurance reimbursement is available for naturopathic care in twelve states.

### Psychedelic facilitators

While the governance of therapeutic substances falls beyond the present work’s scope, the field of psychedelic facilitation—that is, the supportive accompaniment of persons using psychedelics for therapeutic purposes—represents a rapidly-changing area of law and policy in the US. As such, it is important to note that most ‘natural’ and synthetic psychedelic medicines (e.g., ayahuasca, LSD, MDMA, peyote, psilocybe mushrooms and psilocybin etc.) are federally-restricted in the US as ‘Schedule 1’ substances, legally designated as having a high abuse potential and no recognized medical usage. As such, both possession and therapeutic usage thereof are generally considered illegal, though a policy trend toward decriminalization—an issue beyond this work’s purview—is underway across the country.

Ketamine, a synthetic medication that exerts psychedelic effects when administered in lower doses than typically used for its (approved, ‘on-label’) application as an anesthetic, is federally-approved by the US government for medical usage. As such, some licensed health professionals (e.g., psychiatrists, medical doctors) may legally prescribe and administer ketamine ‘off-label’ to treat mental (and other) health conditions. Because off-label uses of pharmaceutical drugs are neither unusual nor outlawed in the US, we will not elaborate further on the medico-legal context for low-dose ketamine’s usage as a psychedelic medicine. Ketamine aside, there are two types of exceptions to the US federal prohibition on psychedelic-assisted therapeutic facilitation that warrant comment.

#### Religious exemptions

There are currently three distinct religious exemptions to US federal prohibitions on psychedelic possession and usage, all meant to enable the use of particular psychedelic medicines within Indigenous spiritual contexts, including in a group setting led by community leaders. The 1978 federal American Indian Religious Freedom Act provides an exemption for members of a religious group known as the Native American Church to use peyote in spiritual ceremonial contexts, on Native reservations across the US. In 2006, a Supreme Court ruling affirmed the right of members of another religious group, Uniao do Vegetal, to use ayahuasca within the context of religious ceremony anywhere in the US. In 2009, an Oregon court ruling similarly ruled that members of the Church of Santo Daime in Oregon, may engage in ayahuasca ceremony, a ruling only applicable in the state of Oregon.

#### Psychedelic facilitator licensing

In 2022, the state of Oregon (where possession of psychedelic substances has been decriminalized) became the first US jurisdiction to legalize the therapeutic application of psilocybin (‘magic mushrooms’ and related products) for mental health purposes for persons aged 21 and older. To this end, the state has begun four-fold process of licensing: a) practitioners of psychedelic facilitation, along with b) related manufacturers, c) laboratories, and d) legal facilitation sites. Persons eligible to become Licensed Psychedelic Facilitators in Oregon must: be at least 21 years of age; be resident in Oregon; have completed a secondary school diploma; undergo a criminal background check; complete a state- approved program of psychedelic facilitation training lasting at least 120 hours and meeting defined core requirements; and pass a related examination administered by the state.

Two other states–Texas and Utah–have recently passed laws (in 2021 and 2022 respectively) to enact studies related to possible future implementation of similar programs.

### Safe harbor laws for traditional and complementary medicine practitioners

Ten US states have enacted statutory provisions that provide ‘safe harbor’ to permit otherwise-unlicensed practitioners of traditional and complementary medicine approaches to provide (for a fee, or on a volunteer basis) a wide range of therapeutic practices to members of the public, without risking charges of practicing medicine without a license. Two such provisions (in Idaho and Oklahoma respectively) are articulated as statutory exemption to state Medical Acts, which otherwise restrict the practice of medicine to licensed physicians. Idaho’s law, the nation’s first ‘safe harbor’ medical law (enacted in 1976), provides a statutory model that is subsequently followed in seven other states. This model has two basic components, the first of which involves characterization of therapeutic approaches not meant to be otherwise subject to state medical law. The second component involves a ‘disclosure’ that requires the unlicensed health care practitioner to provide a written disclosure to their clients which includes (at a minimum) the practitioner’s name and contact information, type of therapeutic practices offered, and the practitioner’s qualification to practice. Some state disclosures are constituted as a ‘bill of rights’ for clients; some also include additional stipulations about confidentiality, fees, liability insurance, the client’s right to seek care of their own choosing, and/or accommodations for persons speaking languages other than English and those who do not read. In all cases, clients are required to sign (or otherwise document acknowledgment of) these disclosures.

Specific ‘exempted’ therapeutic approaches vary by state. Idaho, for example, indicates that religious and family healing practices, as well as general health advice and recommendations for natural health products (e.g., herbs, supplements) are exempted. California, by contrast, simply indicate an exemption for any practice that does not encroach on particular practices restricted to other licensed health care professionals; Louisiana exempts an umbrella category of practices characterized as “Lifestyle Modifications”, defined as a "broad domain of traditional or homeopathic healthcare practices and other complementary health practices and services" otherwise unlicensed in the state. Other states (e.g., Colorado, Rhode Island, Minnesota, Nevada, New Mexico) elaborate longer lists of specific health practices that unlicensed practitioners may provide (e.g., acupressure, homeopathy, lifestyle modifications, Gerson and colostrum therapies, mind-body practices, aromatherapy, culturally-traditional healing practices).

Three states with licensing laws for naturopathic doctors (New Mexico, Minnesota, Rhode Island) include ‘naturopathy’ in their list of exempted practices, indicating that practitioners of naturopathy who are not licensed as naturopathic doctors may continue to hold themselves out (and practice) as naturopathic practitioners, as long as they do not use state-restricted titles. Colorado uniquely stipulates that colon hydrotherapists are not exempted except if they complete specified certification trainings. In the state of Arizona, where medical doctors may be uniquely licensed as ‘homeopathic physicians’ a limited statutory exemption exists to permit the practice of homeopathy by lay practitioners who are not otherwise licensed as ‘homeopathic assistants’ to homeopathic physicians.

## Discussion

As the WHO rightly observes, the US currently has “no national plan with respect to the integration of T&CM into mainstream health service delivery” [3]. However, as the present work documents, individual US states and territories have implemented over three hundred pieces of legislation that license or otherwise statutorily govern (albeit in a patchwork manner) several groups of T&CM practitioners. Furthermore, there exists considerable government-funded reimbursement for a subset of T&CM care across the country, both at the federal and state-specific level. In what follows, we contextualize our study findings within the broader US health professional landscape.

Because health professionals are governed at the state and territorial level across the U.S., and each jurisdiction creates its own unique governance frameworks for such professionals, licensure laws, restricted professional titles, and scopes of practice vary across the country for some T&CM professional groups. That being said, a few titles—including Chiropractic Doctor, Licensed Acupuncturist, Naturopathic Doctor, Licensed Massage Therapist, Certified/Registered Nurse-Midwife and Licensed/Registered Midwife—are entrenched in statute across numerous states and have thus become recognizable national monikers for their respective professions. Furthermore, professional entry requirements for T&CM practitioners seeking licensure with their respective jurisdictional regulators are, however relatively consistent across the country, with several national, non-governmental professional organizations and accrediting bodies often providing standardized mechanisms for credentialing practitioners. Notably, of the five major T&CM occupations subject to licensure across much of the US (acupuncturists, chiropractors, massage therapists, midwives, and naturopaths), massage therapy is most strongly characterized by variability, both in terms of professional entry requirements and in terms of the types of bodywork practices included in the scope of state licensure. Further, just two of the US’s ‘big five’ T&CM occupations have implemented multilingual credentialing examinations—massage therapy (English and Spanish) and acupuncture (English, Chinese and Korean). While scopes of practice for these ‘big five’ are partially-harmonized across licensing jurisdictions, contested practice areas remain (e.g., Chinese herbal medicine as a component of licensed acupuncturist practice; Asian bodywork therapies, reflexology, and structural integration (‘Rolfing’) for massage therapists; and, for chiropractors and naturopaths, pharmaceutical drug prescribing, minor surgery and injection/intravenous therapy rights).

Similarly, within a US health systems context wherein T&CM care is generally paid for on an out-of-pocket basis (and in some cases reimbursed by private insurance plans), government-subsidized insurance coverage for T&CM practitioners and practices exists in a disparate patchwork across the country. The evidenced value of non-pharmacological therapies (including multiple T&CM approaches) for treating pain has led to increased government-subsidy for such services in recent years. However, notable barriers to T&CM practitioners being able to access such benefits for their patients persist. For example, while US federal Medicare officially reimburses the limited provision of acupuncture to treat pain, licensed acupuncturists may only be reimbursed if employed by eligible Medicare providers (e.g., medical doctors, nurses); similar barriers are evident with respect to massage therapy reimbursement, where it exists. Limited insurance reimbursement for T&CM practitioners remains a notable barrier to these professionals’ economic well-being, in light of heavy student debt loads.

Importantly, the country’s major T&CM professions are neither equal in size nor equivalent in earning power. Massage therapists constitute by far the largest number of licensed T&CM professionals across the US (with over 300,000 practitioners), complete the fewest training hours required for professional entry, and—in addition to having the most limited access to government reimbursement for their services of the ‘big five’—are the lowest paid, on average of the five ($49,260). Put into context, licensed massage therapists—an occupation that is predominantly comprised of women (86%) earn slightly less (on average) than full-time female-identified workers across the country ($51,226), notably less than similar male-identified workers ($61,180) [127]. For massage therapists as household single-earners, their average annual income falls within the second (lowest) quintile of household incomes across the country. Chiropractors, the second largest group of licensed T&CM professionals in the US (n = 77,000) and a group composed of over two-thirds men, earn almost 65% annually more on average than do massage therapists ($81,240), but less than physical therapists (who earn, on average, $95,620) [128]. Acupuncturists (n = 28,000) and naturopaths (n = 6000), relatively smaller occupational groups comprised of 70–75% women, earn on average almost 45% more than massage therapists. While no income data are available for direct-entry midwives, nurse-midwives earn considerably more per year on average ($114,210) than their non-midwife registered nurse counterparts ($77,600), but still considerably less than US biomedical physicians ($208,000). On the whole it would appear that licensed T&CM professionals in the US who are not also members of a licensed biomedical profession (e.g., nursing) earn less than most conventional biomedical professionals.

While the WHO began its global call for the statutory governance of T&CM practitioners in 2002, the trend toward licensure of such practitioners began many years prior in the US, and continues to unfold and transform the country’s health professional landscape today. The chiropractic profession, for example, has been licensed across the US for over four decades, with early chiropractic licensing laws dating back to the early 1900s. Both massage therapists and naturopathic doctors in some states similarly gained licensure in the early 1900s, but continue to lobby for licensure in other states today. Acupuncturists, by contrast, began to gain state licensure much later, in 1973, but achieved almost-universal licensure more quickly than any other T&CM occupation in the country.

Four noteworthy trends in T&CM practitioner governance worth watching as they unfold across the US in the years ahead involve: a) the increasing integration of T&CM services into health care programs for veterans and active military personnel; b) the licensing of lay persons, mental health and social service professionals as auricular acu-technicians, meant to offer limited-scope ear acupuncture, often for treating mental health conditions, pain, and addictions; c) state registration and reimbursement of birth doulas; and, d) licensing of psychedelic facilitators providing mental health care, as decriminalization initiatives with respect to psychedelic substance possession continue to take hold in many states.

As these and other T&CM regulatory trends advance, it is likely that present work’s findings may require periodic updating. It is also important to note that there exist numerous T&CM practitioner communities across the US that are not presently licensed despite having implemented voluntary, non-statutory, and community-based governance mechanisms. Such practitioner communities include Indigenous healers, faith and energy healers, herbalists, yoga therapists, mindfulness educators and others, the governance of whom falls beyond the present work’s scope [27].

Regardless, this study notably contributes, for the first time, a comprehensive, scholarly, nationwide account of the extensive statutory governance and government reimbursement of T&CM practitioners across the US. This account will prove useful for regulators and policy makers, clinicians, educators, students and others seeking to understand the place of non-biomedical therapeutics in the US health care landscape. It will provide an important foundation for future scholarly analyses of this range of policies, whether in terms of implementation successes and challenges, or regarding the impacts on practitioners, patients or health systems more broadly. This study may also serve as a model for similar environmental scans of T&CM practitioner governance in many countries across the globe, where such reporting is currently lacking.

As this work shows, statutory governance of this field may take a range of forms beyond national T&CM integration strategies. Despite the WHO’s emphasis on such national plans, it is critical that other forms of policy-making relevant to practitioners and practices be rigorously documented to foster a fulsome understanding of the state of this field globally. Ultimately, this work, and others following its example, set the scene for more rigorous international reporting and analysis (whether by the WHO or others) regarding this understudied field of health practitioner governance worldwide.
